# Early animal evolution and the origins of nervous systems

**DOI:** 10.1098/rstb.2015.0037

**Published:** 2015-12-19

**Authors:** Graham E. Budd

**Affiliations:** Department of Earth Sciences, Palaeobiology Programme, Uppsala University, Villavägen 16, Uppsala 752 36, Sweden

**Keywords:** nervous system evolution, Ediacaran, Cambrian explosion, environmental change, homoplasy

## Abstract

Understanding the evolution of early nervous systems is hazardous because we lack good criteria for determining homology between the systems of distant taxa; the timing of the evolutionary events is contested, and thus the relevant ecological and geological settings for them are also unclear. Here I argue that no simple approach will resolve the first issue, but that it remains likely that animals evolved relatively late, and that their nervous systems thus arose during the late Ediacaran, in a context provided by the changing planktonic and benthic environments of the time. The early trace fossil provides the most concrete evidence for early behavioural diversification, but it cannot simply be translated into increasing nervous system complexity: behavioural complexity does not map on a one-to-one basis onto nervous system complexity, both because of possible limitations to behaviour caused by the environment and because we know that even organisms without nervous systems are capable of relatively complex behaviour.

## 1. Introduction

The origins and diversification of the animals, a series of events that became manifest in the so-called ‘Cambrian explosion’ of *ca* 540 Ma, must necessarily be intimately tied into the evolution of their important organ systems. Of these, the nervous system must be considered to be of extreme importance, not only because of its universality among animals apart from sponges and placozoans, but also because of the role it plays in coordination, sensing and indeed many other aspects of the life of an animal. Until recently, the idea of the fossil record yielding direct evidence for the presence and nature of the nervous system seemed fanciful, but in the past few years, several direct and indirect lines of evidence have suggested this may be the case (e.g. [1–3]). Nevertheless, evidence for the evolution of the nervous system for the vast majority of animals, and with it the vexed questions of the potential homology of important structures such as the mushroom body across the phyla, must rely largely on more indirect evidence such as phylogenetic reconstruction and comparative anatomy. These endeavours are themselves somewhat hampered by a series of as yet unresolved methodological disagreements, most notably about the resolving power that phylogeny has in such instances, a dispute that essentially boils down to the question of whether or not pronounced similarity can be regarded as a definitive test for homology, irrespective of phylogenetic position.

The fossil record can itself, of course, with the limited exceptions mentioned above, provide relatively little information about the evolution of the nervous system. Nevertheless, it can contribute in two ways: (i) from the important evidence of the early fossil record and (ii) from the body fossil record that can on a phylogenetic basis constrain the timing and perhaps even the ecological basis of the evolution of early nervous systems. In this essay, I explore the somewhat subtle data that may bear on these issues, with an emphasis on phylogeny on the one hand and the importance of the changing Precambrian world on the other.

## 2. The origin of nervous systems: a phylogenetic perspective

From any phylogenetic perspective, the first issue to be addressed in any general consideration of the origin of the nervous system is whether or not it had one or more separate origins. It has been traditional to regard nervous systems as having evolved once only, at the base of the so-called Epitheliozoa (i.e. Ctenophora, Cnidaria and Bilateria)—essentially all of the animals apart from the sponges. However, this simple view has come under attack by recent phylogenetic reconstructions that recover the Ctenophora as sister group to all other animals. This raises the question in an acute form: did the nervous system evolve once, only to be lost in the Porifera, or has it evolved twice, once in the Ctenophora and once in the Eumetazoa (=Cnidaria plus Bilateria)? Both the phylogenetic reconstruction and its implied consequences have been debated quite extensively in the literature, but little consensus has been reached [4–7].

**Figure 1.**
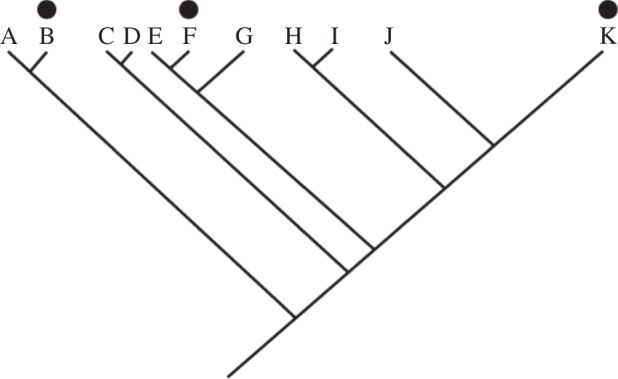
The optimization problem. If a complex and very similar character such as a brain (marked by the black circle) is shared by taxa B, F and K, can it be considered to have evolved at the base of the tree and lost (in a non-parsimonious way) in the clades of A, C–D, E, G, H–I and J, or should it be considered a convergence between B and K?

The origin of the nervous system raises a live issue about the determination of homology, which is the classical problem of similarity versus phylogenetic distribution (figure 1). On the similarity side, are molecular similarities or differences enough to decide homology? If such features are taken as *decisive* in determining homology, then it follows that phylogeny distribution of such features has no real role in determining homology, but rather, provides a framework onto which a pattern of gain and (usually) multiple loss can be mapped. In effect, the idea that homology can be detected by inspection of (molecular) complexity leads to a pronounced type of ‘Dollo’ evolution, where characters are only gained once and then lost but never re-emerge [8], with a difference that it is not just ancestral states that cannot be homoplastic.

One of the promises of the field of ‘evo-devo’ was indeed to provide a secure basis for determining homology based on developmental gene expression, but this proved to be far from reliable, if only because (as per the premise of the field), development evolves [9,10]. Without the invocation of phylogeny, therefore, the question of homology inevitably becomes a subjective debate about how or what sorts of similarity are important or critical. Various attempts at quantifying homoplasy have been made (e.g. the classic work of [11]), but of course, unless one considers loss to be simply a type of homoplasy (e.g. an insect clade that loses its wings can also be considered to have re-evolved a plesiomorphic, eyeless state (cf. [12])), a convinced Dollo evolutionist of this type is always able to consider any pattern of character incongruence to be the result of loss and not convergence. Thus, despite determined attempts to catalogue convergence (e.g. [13]; cf. [14]; weboflife.com), the basic problem of interpretation of character incongruence remains, although clear cases of re-emergence of features do exist [15,16]. The implications of considering evolution in this way can be quantified, however, with a classic example being a discussion of the supposed re-evolution of wings [17] within phasmatids (i.e. stick insects). The discussion of [18] shows that only a relatively small excess ratio of loss rate over gain (which is known to be the case based on the many documented cases of wing loss in insects) is enough to account for the phylogenetic distribution of wings without implying re-evolution of this complex character (cf. [19]).

Questions of loss and gain have been made more problematic by recent explorations of the very meaning of homoplasy (e.g. [20]), with the implication that parallelisms (in the sense of phenotypic change being driven by underlying and fixed genetic structures) are much more common than previously considered by the classical literature on convergence. This idea, another central feature in much of modern evo-devo, implies that internal structure drives phenotypic change at a deep level and is, as is sometimes admitted [20], an at least partly anti-adaptationist programme. In the context of the very general features being considered here such as the nervous or visual systems, however, it is not at all clear that such features can be regarded as adaptations, because these ‘finished products’ (the compound characters of [21]) did not arise as single advantageous evolutionary events. Rather, they are the (current) endpoints of a *series* of adaptations and functional shifts, many or all of which had perhaps little to do with their present function. Therefore, one can both agree with an anti-adaptationist view of such features while simultaneously affirming the fundamentally adaptive basis of their complex origins. Animals with nascent nervous systems surely did not encounter a single ecological challenge that enabled the evolution of more complex forms: rather, they were placed in a series of contexts, each of which tended to stimulate (or even depress) the development of the system. The current nervous system of, for example, bilaterians, is thus presumably the endpoint of a series of balanced and potentially contradictory demands for locomotion, sensing, reproduction, etc. If a functional analysis of such demands (such as fig. 4 of [21]) could be made, then this may be an important milestone in understanding the origin of modern nervous systems.

It is in this whole life-history context that the question of homology or homoplasy of nervous systems should be considered, and perhaps it is this holistic approach that offers the best chance of resolving the question (for previous discussions of this subject, see e.g. [22,23]). Consider, for example, the nervous system of a rotifer [24,25] and a vertebrate with the aim of resolving the question of whether or not the rotifer once had such a complex nervous system as the vertebrate but has now lost it. One can of course plot out various nervous systems onto an animal tree and optimize their internal character states using various assumptions. However, if one is persuaded that very basal features of nervous systems must be homologous across the phyla, then the ecological context of the now-vanished complexity must have once existed within the stem-group of the simplified group in question. Here one can consider the insights of, for example, Olive [26] who recognized that such important features of animals do not exist in isolation from each other, but rather come in complementary sets. Thus, animals that are large also tend to have body cavities, storage of gametes and external fertilization with primitive sperm structure, etc. If the appropriate set of correlates of complex nervous systems could be established within this framework, then for the stem-group of, say, the rotifers to have once possessed such complexity would have important implications for other general features, and these might be detectable either in the fossil record or, more simply, be also inferable from the tree. To put this point in its most simple and stark form: some rotifers are stationary organisms with (all things considered) relatively few neural demands, and unless their ancestors had ecologies that can be inferred or observed to have been considerably more complex, it is extremely unlikely that they simply ‘happened to’ possess more complex nervous systems too (one can in this context consider tunicate ontogeny, for example, which on adopting the lifestyle of such a rotifer greatly modify the more phylogenetically informative nervous system of the larva [27]).

This rather preliminary discussion cannot, of course, resolve the vexed problem of the degree of nervous system homology within animals, but is intended to place it within a broader context than *simply* distribution on the tree versus degree of similarity. One way of assessing this context is via the fossil record, which may record aspects of deeper hierarchical levels of animal phylogeny than currently represented by any living animals, and it is to this that I now turn.

## 3. The trace fossil record and its early manifestation

Trace fossils are essentially the remains of biologically mediated interactions of organisms with sediments. There are many ways in which an organism can leave some sort of trace in the sedimentary record, for example, by an empty shell bouncing along the sea floor leaving a series of indentations. However, for such a remain to qualify as a trace fossil in the strict sense, it must both reflect the anatomy of the maker in some way, and also represent the traces of *biological activity*, i.e. behaviour (for the taxonomy of traces in the broadest sense, see e.g. [28]). Although a wide range of types of trace fossils exist (e.g. the theoretical trace of flying left by an organism whose wing tips just brush the surface of a mud flat as it flies low across it), for most purposes trace fossils can be considered to consist of *tracks* (the remains of walking or crawling across the surface) and *burrows* (the remains of digging into the sediment, either to form a permanent residence or to move from one place to another). Around the central core of recognized tracks and burrows exist a rather large penumbra of both *dubiofossils* (structures that may have an organic origin, but about which strong doubts exist) and *pseudofossils* (structures that appear to be organic in origin but in fact are known to have been formed in some sort of inorganic way). The early trace fossil record is replete with examples of just such cases (the best review of the Proterozoic trace fossil record remains [29]), which one by one have been shown to be spurious, with the exception of certain traces from relatively close to the Ediacaran–Cambrian boundary. Accepted Ediacaran trace fossils are largely archetypically simple meanders that were evidently formed close to the sediment–water interface [29] and which apparently lack true branches: the oldest dated examples of these simple traces are from the White Sea area and are younger than about 560 Myr [30]. More recently, a relatively convincing case has been made for trace fossils being present in the Mistaken Point assemblage of Ediacaran fossils from *ca* 565 Ma [31] and these must be currently taken as the oldest known examples of organisms disturbing the sediment. Two other recent examples, i.e. [32] and [33], can be questioned on the grounds of either age (the fossils may be Permian rather than Ediacaran) or authenticity (the fossils may be body or protist rather than animal trace fossils of some sort). One further trace fossil that has been much discussed recently is that associated with the problematic *Kimberella* in the Ediacaran biota [34]; this has been suggested to be the work of a mollusc-like organism raking through the sediment with a radula. What one thinks of this idea is rather dependent on the affinities of *Kimberella* [35]*.* While there has been in recent years a growing tendency to regard it as a mollusc [36], the material from the White Sea in particular [37,38] must cause some pause, and we have argued that its affinities must currently remain highly unclear, although it is clearly an animal, with perhaps bilaterian affinities [35]. If *Kimberella* did turn out to be a (stem- or crown-group) mollusc, then clearly some important assumptions I make in this paper would have to be re-examined, for it would imply that a major bilaterian radiation would already have taken place by *ca* 555 Ma, with a presumably wide range of nervous systems (complex, simple and secondarily simplified) having already appeared. Confirming the identity of *Kimberella* must thus remain an urgent task for clarifying the history of nervous system evolution.

Leaving aside the *Kimberella* traces then, what can be said about the Ediacaran trace fossils in general? First, they show a degree of change throughout the period: the oldest are the rounded structures seen in the Mistaken Point biota; then come the typical gently meandering traces, some with levées (i.e. banks of sediment either side of the trace channel demonstrating that the maker was pushing its way through the sediment and thus displacing the sediment to each side); then come, towards the end of the Ediacaran the first more complex traces such as simple treptichnids that start to show a more three-dimensional structure, followed, almost exactly at the base of the Cambrian (indeed, it is meant to be defined by its appearance) by the canonical *Treptichnus pedum*.

**Figure 2.**
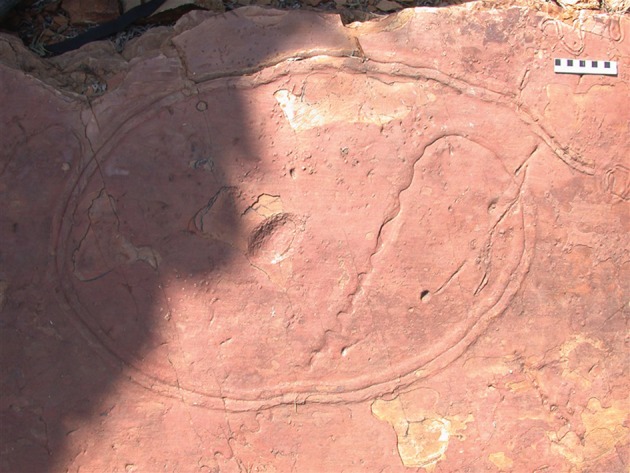
A large specimen (in the field) of the lower Cambrian trace fossil *Plagiogmus arcuatus* from the Uratana Formation near Alice Springs (photograph kindly provided by Sören Jensen). The organism making the trace made a large loop and on completing it began to burrow down into the sediment, with its siphon-like structure rocking from side to side to make the sinusoidal pattern. Such a complex trace is extremely likely to be of (unknown) bilaterian origin. See [35] for an analysis of its mode of formation. The oval structure in the centre is an eroded ripple suggesting a shallow water environment. Scale bar, 5 cm.

The contrast between the usually small (at most a few millimetres across) and simple trace fossils of the Ediacaran and those of the early Cambrian sediments is striking. For example, in the earliest Cambrian Uratana Formation of Australia, traces such as *Plagiogmus* are known, which have considerable complexity in terms of both overall morphology and internal structure (figure 2), structures that have led to considerable speculation on the sort of behaviour and maker involved [39–41].

Cambrian traces are thus clearly more complex than Ediacaran ones, and this must have some implications for the nervous systems of whatever animals were making them [42]. However, the relationship between trace morphology and nervous system complexity is—to say the least—an obscure one. After all, single-celled eukaryotes such as foraminiferans are capable of exploring surfaces and (at least potentially) leaving quite complex traces (e.g. [43]), as are placozoans and the larvae of sponges [42]. Modelling of quite complex trace fossils has shown that they can be generated by a very small set of instructions (e.g. [44]) or a neural network system (e.g. [45]) that could plausibly reflect cues from chemical gradients rather than complex nervous systems. On the other hand, the large and complex *Plagiogmus* of figure 2 could hardly have been made by anything other than a bilaterian. Our confidence about this seems to be largely based, not on a *theoretical* understanding of minimal nervous system complexity for a given behaviour, but rather an observed *empirical* correlation. In other words, the reason one might think that *Plagiogmus* is made by a bilaterian is surely partly that only bilaterians today make trace fossils of such complexity. Furthermore, even the fairly simple trace fossils found in the latest Ediacaran are more or less identical to trace fossils found in the record today that we know are made by bilaterians, and it would take a particular type of special pleading to argue that some wholly other group of organisms in fact produced them. If this assessment is correct, then our ability to trace the evolution of nervous systems in the fossil record must largely rest on phylogenetic considerations, and not necessarily on simply ‘reading’ nervous systems from fossilized behaviour. One possible way of doing this might rely on being able to distinguish behaviour based on internal versus external representations of the environment, with the implication that only organisms with a complex nervous system would be capable of producing the latter. However, this in turn would imply the ability of animals with complex systems to be able to form internal representations, and it is not at all clear that even expert navigators such as insects in fact have this ability [46].

The appearance of definitive trace fossils from about 560 Ma onwards is thus likely to reflect the presence of bilaterians from that time onwards, as today only bilaterians make such traces; we can thus also say that whatever nervous systems characterized early (i.e. stem-group) bilaterians were also present by this time. Similarly, one can also say from the pure geometry of animal phylogeny that stem-group cnidarians, sponges and ctenophores—and whatever nervous systems they possessed—were also present.

This minimalist reading of the fossil record would be broadly agreed on—no-one seriously thinks that there were no stem-group bilaterians in the Ediacaran, for example. But what of more maximalist readings (e.g. [47,48])? Molecular clock estimates continue to place animal origins considerably deeper than anything the fossil record might indicate (e.g. [49]). This would imply, for example, that complex nervous systems such as the brain of protostomes (and possibly of bilaterians as a whole) evolved as a result of what must be considered to be wholly cryptic processes [50]. Nevertheless, functional similarities across the clades in question must suggest the opposite—that we indeed have some hints about why at least some components of such nervous systems evolved—whether it was for reproduction, location of prey or coordination of locomotion. Despite the evidence from molecular clocks, the idea of all these complex ecological challenges arising in microscopic and planktonic organisms that would leave no trace on the benthos continues to seem highly unlikely (e.g. [51]). The increasing complexity of trace fossils from the late Ediacaran through to the Cambrian must also be seen in this light, and this raises an interesting and subtle issue: does trace fossil complexity reflect ecological opportunity or morphological and physiological limitation? For example, is the reason that Ediacaran trace fossils are simple and horizontal because they were burrowing close to the undersurface of algal mats, and because of a hostile and anoxic environment at depth [52], or because the organisms that made them were incapable of more complex movements? Some evidence that could bear on this issue comes from the trace fossil record of the invasion of lacustrine environments later in the Palaeozoic (e.g. [53,54]), which shows a somewhat similar progression of first rather simple and two-dimensional traces into more complex forms as metazoans overcame what was presumably a challenging environment for them. Nevertheless, though striking, this similarity does not exhaust the issue, as the earliest traces of these later terrestrial environments already show a degree of complexity (e.g. in the presence of *Rusophycus*-like forms) that goes considerably beyond that of the Ediacaran. A similar pattern can be seen in the re-radiation of trace-makers after the devastating late Permian extinction (e.g. [55–57]), which may be mediated by widespread anoxia.

## 4. Nervous system evolution and environmental change: the Ediacaran view

The view that animals (and especially animals likely to have complex nervous systems) arose in the Ediacaran is one that I have long defended (e.g. [51,58]). The alternatives, based on molecular clock evidence that places their origins well before in the Cryogenian or earlier (e.g. [49]), lack direct evidence from the fossil record, and continued searches for convincing crown-group members of any animal phyla before the very end of the Ediacaran (e.g. [59]) have yet to yield results (cf. [60]). The context for nervous system evolution is thus likely to be the complex changing ecology and environment of the Ediacaran period of *ca* 635–540 Ma [61]. Although our knowledge of this period remains relatively fragmentary, it is clear that at several levels it was an era of exceptional change, with, for example, major continental rifting and collision taking place (e.g. [62]) and striking shifts in oceanic and atmospheric composition. How these changes might be in detail related to the rise of the animals is of course beyond the scope of this essay, but nevertheless two major arenas of change—both ecologically mediated—stand out: the planktonic environment of early larvae, and increasing heterogeneity in the benthic environment.

### (a) Larval nervous systems and the planktonic environment

Although Monk & Paulin [42] propose that the origin of ‘spiking’ neurons was the result of increased ecological pressure to develop rapid predatory behaviours, an alternative view is provided by Jekely [63], who proposed that nervous systems developed via neuronal control of phototaxic swimming ciliary systems in eumetazoan larvae (see also [4] and [50] for broader perspectives on nervous system origins). An interesting aspect to this latter possibility would be the transition from the so-called ‘green’ to ‘blue’ ocean of the Precambrian–Cambrian transition. Although oceanic conditions in the Precambrian must remain rather unclear, it is likely that the export of primary production in the photic zone to the benthos was considerably less efficient than it is today, as it is facilitated by the clumping effect of the mesozooplankton [64,65]. It is thus reasonable to think that both particulate and dissolved organic matter were present in greater concentrations in the water column than they are today. This would have had a considerable effect on the attenuation of light in seawater, with the dissolved organic carbon (DOC) having the effect in particular of shorter wavelengths being absorbed exponentially more than longer ones (e.g. [66], their fig. 8). Phototaxis in early larvae would thus likely to have been tuned, presumably by their utilization of opsins [67,68], to longer wavelengths than is appropriate in typical open-water modern environments, although analogous modern environments might provide useful insights into what sort of environmental challenges they faced (see e.g. [69]); the use of polarized light would also have been limited [69,70]).

### (b) The elaboration of benthic ecology

In the palaeobiological literature, the role of predation in driving elaboration of body plans generally (e.g. [71]) and nervous systems more specifically [42] has loomed large. However, predation requires both prey and a set of specific adaptations such as (for example) mobility for many predators. While I do not deny the role of predation in evolution, the specific features required for it to function effectively are to have arisen pre-adaptively in other ecological contexts [21]. We have elaborated elsewhere [35] a theory of major bilaterian innovation taking place within the known ecological context of the late Ediacaran, i.e. that provided by the enigmatic ‘Ediacaran’ taxa such as *Spriggina*, *Charniodiscus*, etc. (cf. [72,73]). These taxa, despite their notoriety for obscurity and the plethora of sometimes extraordinary suggestions for their affinities (ranging from vertebrates to lichens), are very likely to represent various stem-group branches of early animals (cf. [35,51,74]). It follows that in this view of early bilaterian (and indeed eumetazoan) evolution, centralized nervous systems arose within relatively large organisms that likely already carried out a range of more or less complex functions, rather than arising in small animals that were enabled to perform more functions by their appearance. Given that all the evidence we have suggests that the early bilaterians arose within such a setting (and early trace fossils are invariably found in or close to beds with Ediacaran body fossils in), it follows that the evolutionary context for the expansion of bilaterian nervous system construction was likely provided by their interactions in such communities, one of which (eventually) was to be predation. It is notable that although total-group cnidarians and ctenophores must have branched before the beginning of the Cambrian, it is not until the Precambrian–Cambrian boundary that easily recognizable and potentially crown-group members of these clades appear. This is of potential importance in considering their distinctive prey-capture mechanisms (colloblasts and cnidocytes): before the evolution of the zooplankton, it is not clear what large prey these animals would have been living off. The presence of at least nematocyst-like structures in dinoflagellates [75] has even led to the tantalizing suggestion that cnidarian prey-capture mechanisms are derived from once-symobiotic dinoflagellates [76]. In any case, it is not unreasonable to think that the evolution of the crown-groups of these clades was driven by the evolution of the later bilaterians, an event that must have caused a cascade of evolutionary events in many other ecosystems [77]. Finally, although the role of oxygen levels in animal evolution has been recently questioned by various authors [64,78,79], if oxygen levels really did markedly rise at the end of the Ediacaran (either for geological or ecological reasons), then this may have been permissive for the elaboration of energetically expensive nervous systems within emerging bilaterian clades.

## 5. Discussion: the reconstruction of early nervous system evolution

In this essay, I have tried to sketch out some of the methodological and empirical challenges that face the recovery of the early stages of nervous system evolution. These include (i) trying to calibrate the use of phylogeny in order to test homology statements, (ii) placing this evolution in the right time (and thus geological and ecological setting) and (iii) exploring the various and perhaps contradictory pressures for nervous system evolution within such settings. The first of these issues is really one that has infiltrated many aspects of ‘evo-devo’ discussions, and unfortunately it is not enough to simply use an unvarnished cladogram as weaponry against claims of deep homology: the possibility of Dollo-style multiple loss over even a few gains is always possible and indeed has some empirical support (e.g. the case of wing loss in insects). For proper scientific and testable hypotheses of homology, something more is needed than subjective arguments about how ‘similar’ some structures are at either the structural or molecular level on the one hand, and how many times more likely loss is than gain on the other. While we as yet have little insight into the relevant data that might help solve this, it seems likely that we will have to ‘go the bloody hard way’ [80] and move a long way beyond either of these extremes before we have a satisfactory method of resolving these issues (for one possible but now somewhat dated attempt involving the evolution of the arthropod ectoderm, see [81]). The second issue is a straightforward one of an apparent data conflict between the molecular clocks and the fossil record; I have argued in other places that the possibility of animals undergoing an enormous radiation while leaving no trace in the fossil record is wildly implausible. Hence, the most reasonable time to look for when animals (and their nervous systems) evolved is when the fossil record suggests they did, i.e. during the second half of the Ediacaran period. While this was a period of intense change, both ecologically and geologically, the immediate context for nervous system evolution is likely to have been provided by the ecological context of the early animals, and I briefly touch on two such—in the plankton, for the challenges facing (some) larvae, and in the benthos for (some) adults. The best evidence we have for early nervous system remains the Ediacaran to Cambrian trace fossil record, but its increasing elaboration across the boundary cannot be *simply* read as increasing nervous system complexity, as ecological opportunity also seems to play a role in determining trace fossil morphology (as partly demonstrated by later recoveries from mass extinctions). As in [51] and [4], it seems likely that complex systems evolved ‘*in situ*’ within large animals that already had the capacity to perform the actions that were then stabilized by and perhaps improved by later innovations, of which one of the most important was, naturally, the nervous system.
